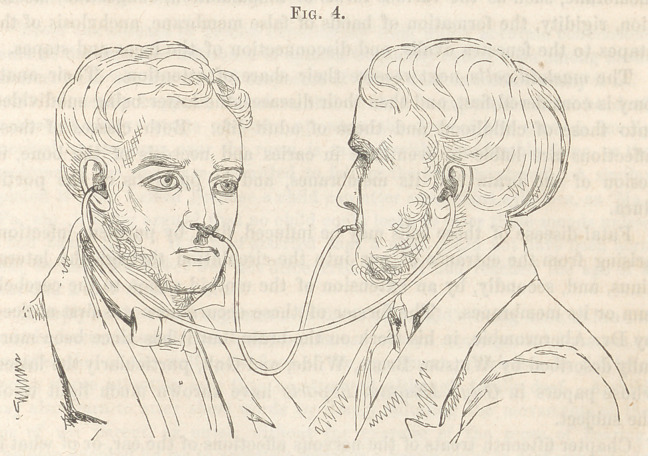# The Diseases of the Ear

**Published:** 1860-09

**Authors:** 


### NORTH AMERICAN MEDICO-CHIRURGICAL REVIEW.

SEPTEMBER, 1860.

### Analytical and Critical Reviews.

ART. I.—*The Diseases of the Ear; their Nature, Diagnosis, and Treatment.* By JOSEPH TOYNBEE, F.R.S., etc. etc. etc. With One Hundred Engravings on Wood. 8vo., pp. 440. Philadelphia: Blanchard & Lea, 1860.

THE work of Mr. Toynbee comprises, within a comparatively small compass, the most valuable practical treatise on the diseases of the ear that the English language can boast of; a work, in fact, which, whether we regard the originality of its matter, the purity of its style, the systematic order of its topics, or the beauty and variety of its illustration, is alike creditable to the author and the profession of Great Britain. The American publishers, who have issued the work *pari passu* with the European edition, deserve the highest praise for their efforts to reproduce the treatise in every way commensurate with its intrinsic merits and the artistic requirements of the age. Messrs. Blanchard & Lea have never issued any work in more excellent taste. The volume is destined to create a sensation on this side of the Atlantic, and will be universally regarded as a worthy successor to the admirable treatise on Aural Surgery by Mr. Wilde, of Dublin, republished by the same distinguished firm six years ago.

There is no man in the "wide, wide world" who has made the diseases of the ear a subject of such profound and long-continued study as Mr. Toynbee, and the result is that he has produced a work which will occupy for ages to come the foremost rank in this department of pathology and practice. For nearly a quarter of a century he has been almost incessantly devoted to the subject, having performed, during this period, an immense number of dissections of diseased ears, treated thousands of cases, embracing all classes of affections, and published upwards of fifty papers, all founded upon personal observations. Never, since the commencement of the profession, was there a man more thoroughly prepared, by previous investigation and elaborate study, for the task of writing a lasting monograph upon any subject. In addition to his long-continued researches, of themselves sufficient to inspire his reader with the most profound confidence in whatever may emanate from such a writer, Mr. Toynbee has lectured for a number of years on aural surgery in the Medical School at St. Mary's Hospital, and has been connected with several establishments as aural surgeon, thus affording him immense opportunities for the observation and study of the maladies and accidents under consideration.

Every one acquainted with the history of aural surgery must be sensible of the extraordinary neglect in which it is generally held by the profession. Even in Great Britain, where medicine is cultivated with so much ardor and success, and where aural science has in late years achieved its greatest triumphs, it would appear that the practice of this department of the healing art is still very much in the hands of the empirics; and in this country our ignorance of the nature and treatment of the diseases of the ear is absolutely proverbial. Nothing, indeed, proves so clearly the low condition of the science of the aurist in the United States as the fact that all our light upon the subject is derived from foreign sources, no native work, founded upon original observation, having yet appeared in relation thereto. Even our periodical literature, so rich in valuable material upon almost every other topic, rarely furnishes anything of interest in regard to the affections of this important organ. For such neglect, which might almost be regarded as criminal, there is, unfortunately, no adequate excuse. It is true, the maladies of the ear are, in comparison with those of some other parts of the body, rather rare; still they are sufficiently common to render them objects of the deepest interest, and hence it should be the duty of every enlightened practitioner to make himself familiar with their nature and management. In fact, his education should not be considered as complete without such knowledge. In our larger towns and cities more especially, where, from the denseness and variety of the population, all classes of aural affections are of daily occurrence, a want of opportunity for observation can certainly not be urged in defense of our ignorance and neglect of one of the most useful and noble studies that can possibly engage the human mind. On the contrary, there is every reason that can be presented on the plea of humanity and science why they should be properly investigated, and the results of our experience be communicated to the profession. From the want of attention to the subject, thousands of persons annually become deaf and wretched simply because the medical attendant fails to make himself acquainted with the nature and treatment of their diseases.

No doubt, one reason of this supineness and ignorance of our practitioners is the intrinsic difficulty of the subject, growing out of the complicated structure of the ear, and the great depth at which this organ is situated; or, in other words, the trouble and loss of time involved in its examination when invaded by disease or assaulted by injury. It is questionable whether any other part of the body, in a morbid condition, demands so much patience and skill for the detection and successful removal of its various maladies as this. Besides, it is well known that the patient himself often opposes to the practitioner serious obstacles toward a successful cure of his case. This remark applies not merely to children, but also, and perhaps even in a greater degree, to grown persons, who too frequently, as our own experience tells us, throw aside the most scientific and skillful treatment under a supposition, which no argument or reasoning can satisfy, that it will not be productive of any ulterior benefit. Doubtless, however, the main cause of this supineness and shameful neglect is the fact that the diseases of the ear meet with so little attention in the lecture-room and in our systematic treatises on surgery. In most of the schools of the present day, certainly in those of this country, aural diseases are either wholly neglected, or they receive the merest passing notice from the teacher. The consequence is that the young graduate, upon setting out in practice, is totally unfit for the treatment of this particular class of affections, and that the cases which fall under his observation are either slighted by him, or, what is perhaps still more lamentable, that they fall into the hands of unscrupulous and designing quacks, bent not upon the patient's cure, but upon a large fee.

We believe it may safely be asserted that there is not, at this time, in this country, a solitary institution conducted upon scientific principles, devoted exclusively, or even in a considerable degree, to the treatment of aural diseases. There is not, so far as we know, a single ward or bed set apart for this object in any of the hospitals in the United States. We have eye-infirmaries, and even eye-wards, in abundance; but nowhere any conveniences for the study and treatment of the affections of the ear, considered as a specialty. What a pity that there could not be some deaf Girard, or Astor, or Lawrence, or McDonogh to found ear-infirmaries in every city of this great Confederacy! What a pity that there could not be such a man at the head of our government as Louis Napoleon, to institute lectureships on aural medicine and surgery in our schools! Republicanism needs, in all matters of science and education, the decision, the independence, and the spurs of a monarchy; the will and the energy of one head.

The history of acoustic surgery was, until recently, a history of the grossest acoustic charlatanry. To recount it would only be to condemn it. Its traces extend back to the earliest records of the healing art, but in these traces we discover only the most profound ignorance. How, indeed, could it have been otherwise? As the ancients were ignorant of the anatomy of the ear, and of diseased structure in general, it would be unreasonable to suppose that they had any substantial or consistent notions respecting its lesions. The knowledge of Hippocrates, Galen, Celsus, Oribasius, Alexander, Aurelianus, Avicenna, Albucasis, Haly Abbas, and other early writers hardly extended beyond that of an ordinary ear-ache. Avenzoar treated otitis with the oil of eggs; and for the cure of chronic noises, Paul of Ægina recommends the use of the syringe with a solution of nitre, vinegar, and honey—fit emblems of the practice of many a charlatan of our own day!

Ambrose Paré, in his immortal work on surgery, speaks of various affections of the ear; among others, of foreign bodies, and of the proper method of extracting them; of tumors, ulcers, preternatural closure of the meatus, the causes of deafness, and the means of meeting the loss of the concha. He also gives a figure of an artificial ear. It is clear, however, from his writings, that his knowledge of the subject was lamentably limited and imperfect; and the same, in fact, is true of all succeeding writers down to a comparatively recent period.

One of the earliest works on aural surgery in the English language was that of Mr. John Cunningham Saunders, of London, issued in 1806, under the title of the "Anatomy and Diseases of the Ear." It was a small monograph, written by a man of science, and by one who had evidently a very respectable practical acquaintance with aural affections. The book, which was chiefly valuable on account of the beautiful delineations of some of the more important diseases of the ear, was soon after reprinted in this city, with notes and additions by Dr. William Price, then one of the surgeons of the Pennsylvania Hospital.

In 1817 appeared the essay of John Harrison Curtis, a shallow, miserable performance, known chiefly by its high-sounding title, and by its having been copied, in great measure, from the treatise of Mr. Saunders. The work was evidently written as an advertisement for business; a practice which is not yet altogether extinct among our British brethren, as we see now and then exemplified in the baser productions of the present day.

Remarks similar to the above are applicable to the work of William Wright, which appeared almost simultaneously with that of Curtis. Devoid of science, it was patronized more by the public than the profession, who regarded its author only as another charlatan so common in every large city.

From this criticism we must exempt the treatise of Mr. Thomas Buchanan, published in 1825, as it evidently possessed some claim to scientific consideration, the author being a surgeon of excellent repute at Hull. The book, however, was an exceedingly small and jejune affair, and exerted no particular influence in awakening the attention of the profession to the study of the diseases and injuries of the ear.

The above works constitute, with the exception of some detached papers in various periodicals, nearly all that had been written on aural surgery in Great Britain up to 1838, when the darkened horizon of this department of the healing art began to show some glimmering of light, mainly through the labors of Mr. George Pilcher, as embodied in his "Treatise on the Structure, Economy, and Diseases of the Ear." It was brought out under the sanction of the Medical Society of London, which awarded to it the Fothergillian Gold Medal, and may justly be regarded as the first attempt, on the part of a British writer, to produce a really scientific work on the diseases of the ear. Besides some original observations, Mr. Pilcher, it would seem, availed himself freely of the contributions of his professional brethren in various parts of the world, especially of those of Dr. W. Kramer, of Berlin, whose able treatise was admirably translated in 1837 by Dr. P. R. Bennett of London. As this work has long been familiar to the American reader, through a Philadelphia reprint in 1843, of the second English edition, it would be needless to consume any further time upon it.

In 1840, Mr. Williams published a "Treatise on the Ear, including its Anatomy, Physiology, and Pathology," for which the author obtained a gold medal in the University of Edinburgh. In 1841, Mr. T. Wharton Jones contributed a highly valuable article on the ear to the Cyclopedia of Practical Surgery, edited by Dr. Costello. Three years after this appeared the little work of Mr. Dutton, of Birmingham, entitled "The Nature and Treatment of Deafness and Diseases of the Ear, and of the Treatment of the Deaf and Dumb." It was speedily republished in this country, and may be considered as a fair outline of aural diseases.

In this enumeration of British writers on the surgery of the ear, we have purposely avoided any reference to the works of Mr. Harvey and Mr. Yearsley, as well as the various papers of Mr. Toynbee, amounting, as we have already seen, to upwards of fifty; because the first are of a questionable character in a scientific point of view, and because the contributions of our author are now incorporated in the admirable treatise before us. Nor is it necessary to make any formal mention of the work of Mr. Wilde, of Dublin, since, through its republication in this country and its translation into some of the languages of Europe, it has earned for its author a world-wide reputation. It was the precursor of a new era in aural science, not merely for Great Britain, but for the world at large. It has put to the blush the effrontery of the charlatan, and thoroughly aroused the attention of the profession to a long-neglected but most important study.

In France there has been no lack of original works on the diseases of the ear. Not to mention particularly the treatise of Duverney, issued in 1683, too early to be of any special scientific value, there are, besides the monographs of Desmonceaux, of Alard and others, published in the latter part of the eighteenth and the beginning of the nineteenth centuries, the magnificent volumes of J. M. G. Itard, issued at Paris in 1821, and marking a new era in French surgical literature. Far from being a mere compilation, the volumes are everywhere enriched by the author's personal observation and experience, the whole constituting a production hitherto without a rival in Gallic aural science, pathology and practice.

The excellent but now almost obsolete essay of M. Saissy first appeared in 1819, as an article in the French Dictionary of the Medical Sciences. It was subsequently remodeled and much improved by its author; and, after his death, a new edition of it was issued by M. Montain, with notes by Dr. Perrin. A translation of Saissy's treatise, with a supplement on the diseases of the external ear, was presented to the American medical profession by Professor N. R. Smith. Of the work of M. Deleau, "Recherches Pratiques sur les Maladies de l'Oreille," the first part of which was issued in 1838, it is only necessary to remark that it is one of the best resumés of aural surgery of the present day in the French language.

In Germany, the most important works on this subject are those of Krukenberg, Ettmüller, Meiner, Rosenthal, Beck, Kramer, Linke, Schmalz, and Frank. Of these the treatise of Kramer, of Berlin, is the best known to English and American scholars, chiefly through the translation of Dr. Bennett, republished in this city soon after its appearance in London. A new and enlarged edition of this work was issued by its distinguished author in 1849. Dr. Charles G. Linke's "Handbuch der Theoretischen und Praktischen Ohrenheilkunde" appeared at Leipsic in 1837 and 1840, in two large octavo volumes: the first, which is illustrated by numerous lithographic plates, comprising an account of the healthy anatomy, physiology, and morbid anatomy of the ear; and the second, of its various malformations and diseases. The monographs of Schmalz, of Dresden, and of Frank, of Würzburg, which are among the latest productions of the German press on this subject, possess very little originality, although they afford the advantage of exhibiting a tolerably full digest of the state of aural surgery and medicine at the period of their publication. The treatise of Schmalz had reached its third edition in 1844; an evidence of its high appreciation among our German brethren.

Of the Italian literature on this subject very little is known in this country, beyond the fact that it is very scanty and unsatisfactory. Excepting some detached papers in medical journals and a few meagre monographs, hardly worthy of mention, nothing has emanated from the press of the land of Scarpa and the great school of which he was for upwards of a quarter of a century the acknowledged head.

America has not yet produced any great work on aural surgery. In 1851, Dr. James Bryan, of this city, published "A Treatise on the Anatomy, Physiology, and Diseases of the Ear," which, as far as we know, is the only attempt to enlighten the profession of this country by one of its own members upon the subject under consideration. The work partakes very much of the character of a compilation, yet it is due to the author to say that it embraces a good deal of original matter, and that it exhibits a clear and well written digest of the existing state of the science of aural medicine and surgery up to the time of its publication. As a safe compend, in fact, it is well worthy of the attention of medical practitioners. Of reprints we have already mentioned those of Kramer, Pilcher, Saissy, Dutton, Williams, and Wilde, the last of which should occupy a conspicuous place in every American medical library.

From this rapid survey of the history of acoustic surgery, it will be perceived that there is rather a dearth than a superabundance of great works upon the subject; or, at all events, that their number is much smaller than in most of the other subdivisions or specialties of the healing art. It is, therefore, with peculiar pleasure that we hail the appearance of the work of Mr. Toynbee, convinced that it will exert a most happy and pervasive influence upon this department of surgical science, not only in Great Britain, the country of its nativity, but also among the author's brethren in the United States, where the book has already taken out its naturalization papers. Written, as before intimated, in a style at once classical and attractive, it discusses the subject of aural surgery and medicine in all its branches and ramifications, and with a degree of *directness* and *assurance* well calculated to secure the confidence and good feeling of the reader; circumstances of no ordinary consideration and importance in a scientific treatise.

The treatise of our author opens with an "Introduction," comprising a brief account of the neglect of the study of the pathological anatomy of the ear, the causes of our ignorance of aural surgery, and the mode of dissecting the ear and of investigating its diseases.

Passing by what Mr. Toynbee says regarding the first three of these points, we are disposed to dwell for a moment upon the latter, as the formation of a correct diagnosis is here, as everywhere else, a matter of paramount importance to the establishment of a rational therapeutics. The mode of investigating the diseases of the ear embraces the following considerations: I. The age and occupation of the patient. II. The state of health, temperament, condition of the pulse, and digestive organs. III. An inquiry into the hereditary character of the disease, or, in other words, as to whether any of the patient's relatives are deaf. IV. The history of the affection; its duration and supposed cause; its former and present symptoms, with an account of the amount of audition; and, finally, the circumstances, mental, corporeal, dietetic, and atmospheric, which tend to aggravate the disease. V. The result of the examination: first, as to the distance at which the ticking of a watch is heard; secondly, the quantity and condition of the cerumen, and the state of the dermis and osseous shell of the meatus; thirdly, the condition of the tympanic membrane, as to whether it is dull or shining, transparent or opake, or more or less concave, along with the state of the "triangular bright spot;" fourthly, the condition of the Eustachian tube, in regard to the perception of sound in the tympanic cavity during deglutition, or during a forcible attempt at expiration, with the nose and mouth in each closed; and, finally, the condition of the mucous membrane of the fauces. VI. The effects of previous treatment. VII. Diagnosis.

Conducting the examination in this systematic manner, the time occupied need not exceed fifteen or twenty minutes; while the result, as far as concerns the diagnosis, must generally be most satisfactory. The author lays particular stress upon the mode of using the watch in these investigations, stating that the surgeon should inform himself as to the distance at which it is usually heard by persons whose hearing power is supposed to be perfect, and that it should be gradually brought toward the ear instead of being withdrawn from it.

In the second chapter, the author, after some anatomical considerations, treats, at some length, of the injuries, malformations, and diseases of the external ear.

The most frequent *malformations* of this portion of the auditory apparatus are, first, an entire absence of the auricle, of which several examples have been recorded by Meckel, Fritelli, Overteuffer, and other observers. In a case which fell under the notice of the late Professor Samuel Cooper, of the London University, and which is narrated at length in his Dictionary of Surgery, the deficiency existed on both sides without any vestige whatever of the external meatus, this passage being completely covered by a prolongation of the common integuments. Notwithstanding this, however, the child could distinguish sounds tolerably well. Secondly, there is occasionally a total absence of the lobule, or this structure may be divided by a fissure into an anterior and posterior portion; or, it may be attached, either partially or wholly, to the side of the head. Thirdly, a deficiency of the helix is sometimes observed, the part being perhaps so small and flat as hardly to deserve to be considered as a distinct process. Fourthly, the tragus and ante-tragus are sometimes malformed; they each may be separated into two portions; or they may be inverted, or drawn permanently inward toward the meatus, thereby partially occluding that tube; or they may be more or less extensively united, particularly at their inferior borders, producing, in effect, a similar obstruction. Fifthly, an enormous enlargement, or unseemly hypertrophy, of the outer ear may be enumerated as another malformation, though its occurrence is uncommon.

The most important malformations of the *auditory meatus* are inordinate contraction of this passage; closure of the outer opening by an extension of the common integuments, resembling an imperforate anus; and, lastly, total absence of the tube. In the latter variety, of which some very interesting examples are narrated by different writers, and of which several cases have been noticed by the reviewer, the abnormity usually coexists with a deficiency of the whole organ, the absence of the outer ear being, apparently, only a part of the general non-development.

It occasionally happens that infants are born with the meatus completely filled with the slimy, caseous matter, which so often covers the general surface, and which is probably derived either from the sebaceous follicles, as a defensive secretion, or from the amniotic liquor. However this may be, if the matter be allowed to remain for any length of time it will almost be sure to cause inflammation, sometimes so severe and extensive as to eventuate finally in deafness.

Instances of *supernumerary ears* are sometimes met with, but they are uncommon. Cassebohm, an old author, relates the case of a child with four ears, situated one above the other, and accompanied each by two temporal bones. In 1858, the reviewer saw at the Clinic of the Jefferson College, under the charge of Professor Gross, an example of four ears, two in the natural situation, and the others, which existed in a very rudimentary state, immediately in front of the tragus, over the temporo-maxillary joint. The redundant parts were removed by excision. Occasionally the supernumerary organs occupy the side of the neck.

The external ear is subject to ordinary *inflammation*, erysipelas, and various cutaneous eruptions; but as these are not peculiar to this region, and do not, therefore, require any peculiar treatment, no further mention of them need be made here.

A very curious form of tumor of the external ear has lately been described, termed by some *hæmatoma,* and by others hæmatocele, and supposed at one time, but erroneously, to be peculiar to insane persons. It consists, as the name implies, in an accumulation of blood in the subcutaneous cellular subtance, between the skin and fibro-cartilage, and is generally caused by external injury, as a blow or fall. The deformity produced by its presence is often quite marked. If the blood is allowed to remain, it speedily coagulates, and is capable afterwards of provoking inflammation. The proper treatment consists in the use of evaporating and sorbefacient lotions, aided, if the case be obstinate, by puncture and gentle pressure.

*Scirrhus* of the external ear is exceedingly rare. Kramer details the particulars of a case of this kind; and a very interesting one came under the notice of the writer some years ago, in a man upwards of fifty, who eventually perished from the effects of the disease.

A *fibro-cartilaginous* growth is now and then seen in the lobule of the ear; its causes are unknown, and it is extremely prone to return after removal, however carefully performed. The writer has met with several cases of it, and he has reason to believe that it is more common in negroes than in white persons, and in females than in males. Boring of the ears, and the wearing of rings probably, at times, excites the disease.

Our author speaks of *gouty deposits* as being not unfrequent in the external ear. We do not remember ever to have met with an example of the kind in our practice, and therefore infer that it is more common in Great Britain than in the United States.

Chapter third is devoted to a consideration of the anatomy and mode of exploration of the *external meatus.* Passing by the former of these topics as possessing little of practical interest, we proceed to offer some remarks upon the latter, as being one with which every aurist should be perfectly familiar.

Three points should claim our attention in the exploration of the auditory passage: first, the effacement of as much as possible of the curve of the meatus; secondly, the slight dilatation of the outer membranous and cartilaginous portion; and, lastly, the free introduction of light. The first two of these objects are best accomplished with the speculum; but the meatus may also be straightened by pressing the tragus forward and pulling the outer ear backward; and we have, in our own hands, generally found this method to answer exceedingly well, although it is certainly not equal to the instrumental inspection. The best light is that of the sun, thrown into the parts in such a manner as to illuminate very thoroughly the tympanic membrane and the whole visible surface of the meatus. In cloudy weather and at night, artificial light may be used, as afforded either by the lamp of Segalas or that of Miller. The former is very simple, but as it can only be used with gas, not so convenient. Miller's lamp, originally suggested by Dr. Chowne, consists of a wax taper, inclosed in a Palmer's spring tube, six inches in length, and provided with a reflector, the apparatus being so arranged as to stand upon a pedestal when not in requisition.

In addition to a suitable lamp, an *aural speculum* is necessary, and the one preferred by our author is of oval shape, as originally described by him in the *London Lancet* for August, 1850, and now in pretty general use in England. The expanded portion of the instrument is somewhat flattened, the flattening being at right angles with that of the smaller extremity, thus adapting it more accurately to the object for which it is intended. Every surgeon should supply himself with from three to four sizes of these tubes. Mr. Toynbee thinks that this form of speculum is decidedly superior to the round, invented by Gruber, and generally, but erroneously, known on this side of the water as the speculum of Mr. Wilde, through whose agency it was first generally introduced into practice. For our own part, we are satisfied that the instrument so long in use among us, and figured in most of the systematic treatises on surgery, is quite equal, if indeed not superior to any other contrivance, that of our author not excepted. Perhaps the only objection to it is that it is not quite so portable.

In conducting the examination, the patient is to be seated as nearly as possible on a level with the surgeon, who, holding the lamp in one hand, should feel and inspect the external ear and orifice of the meatus without the aid of the speculum.

"Having done this, a speculum adapted to the size of the meatus is to be taken in hand, and introduced into the orifice of the meatus, care being taken that the long diameter of each coincides. If the speculum enters very easily, and there appears to be room for a larger one, the next size should be selected, and the orifice fully dilated; for in all cases, the larger the speculum used the greater will be the quantity of luminous rays entering the tube, and the more complete the view of the meatus and membrana tympani. The speculum having been introduced, is to be pressed slightly backward, for the reasons I have mentioned, and then, by means of the lamp in the other hand, the rays of light are to be directed successively on the several walls of the meatus and on the membrana tympani. The size of the different parts of the tube, the quantity, color, and position of the cerumen, if present, should be noted; if absent, the state of the part of the tube in which it naturally exists, and the degree of vascularity of the dermis lining the inner half of the meatus.

"A considerable degree of care is required in the examination of the meatus in the infant and child. The total absence of the osseous meatus in the former, and its very limited size in the latter, should always be borne in mind, or the surgeon, when he introduces the speculum, is apt to press upon the membrana tympani. In many cases it is necessary only to open the orifice of the meatus, when the membrana tympani is at once seen without the introduction of the speculum any further."

In chapter fourth is comprised an account of foreign bodies and of collections of cerumen in the external meatus; two topics discussed with the author's usual ability and practical tact.

It is a singular fact that a *foreign body* in the meatus will occasionally give rise to coughing and even vomiting, which obstinately persist so long as the exciting cause remains in operation. These effects are due, as Mr. Toynbee supposes, and doubtless correctly, to irritation of the auricular branch of the pneumogastric nerve. He refers to two cases, in one of which, under his own care, a patient suffered under an obstinate cough which was promptly relieved by the extraction of a piece of dead bone from the meatus; and in the other, related by Arnold, where a child, affected with chronic vomiting which long resisted all medical means, was effectually cured by freeing each ear of a bean.

For the removal of all rounded solid bodies the author prefers, as does in fact every one else, the syringe and warm water, the fluid being thrown in with some degree of force from a large instrument with a rather long, slender nozzle. Wool, cotton, tobacco, leaves, paper-pellets, and similar articles are frequently more easily extracted with the forceps, or blunt hooks. Insects getting into the meatus sometimes occasion great distress. In general, the use of the syringe, or, if that be not obtainable, the introduction of a little warm water, affords instant relief. The employment of instruments for picking out foreign bodies is often fraught with danger, especially in the hands of ignorant practitioners, from the tendency which the effort has to push the substance farther inward toward the tympanic membrane, which is sometimes severely contused, lacerated, or inflamed in consequence.

The author very properly cautions against interference in the case of foreign bodies when the ear is in a state of inflammation, whether from the lodgment of the substance upon the tympanic membrane or previous attempts at extraction. The appropriate remedies under such circumstances are leeches and fomentations, with purgatives and anodynes.

The remarks on the accumulation of *cerumen* are elaborate and full of practical suggestions, but want of space compels us to pass them by with a few brief comments. The causes of this affection, says Mr. Toynbee, are two: the one depending upon disease of the ceruminous glands; the other, upon secondary and sympathetic disorder of the deeper seated structures, as debility of the auditory nerve, thickening of the tympanic membrane, anchylosis of the stapes, or obstruction of the Eustachian tube. The probability of such a complication may be pretty certainly inferred if, after a collection of this kind has been removed, the hearing power is not wholly, or in a great degree, restored. The causes occasioning such obstruction, not complicated with the affections here enumerated, are: unusual narrowness of the meatus, the effects of cold, the introduction of dust, and the practice of inserting the finger or point of the towel into the ear, thereby pushing the wax farther down. It is obvious that the causes of these various kinds of obstruction should always be carefully inquired into, and, if possible, rectified.

"*The symptoms* of an accumulation of cerumen are: sudden deafness, often following a cold by which the dermis is tumefied; bathing or the introduction of water into the ear. This deafness is often better in the morning; is increased by the movements of the jaw during mastication; and often disappears as suddenly as it came, with a cracking sound in the ear. The cause of the sudden appearance and disappearance of the deafness is the movement of the mass of cerumen: when it is so placed as to allow sonorous vibrations to pass between it and the wall of the meatus, the hearing returns; but when it again comes in contact with the meatus, the deafness recurs. Oftentimes a feeling of fullness in the ear is complained of; not unfrequently there is singing and giddiness, and sometimes considerable pain."

As an accumulation of ear-wax, especially if large and long retained, must always be a source of mischief, besides occasioning more or less deafness, it becomes an object of paramount importance in every case to effect its early removal. The best and most efficient means for accomplishing this is the syringe and warm water, which will always thoroughly dislodge even the hardest masses, though the operation sometimes requires to be several times repeated before the object is fully attained. The syringe recommended by the author holds three ounces and a half, and is furnished with two rings, so that it may be held firmly in the right hand, and leave the left at liberty to hold the ear, which should be drawn well backward in order to straighten the tube. If the wax be very hard, a weak alkaline solution may be employed to soften it before recourse is had to the syringe. Finally, the injurious effects of accumulations of this kind are illustrated by several very interesting and instructive cases from the author's practice.

The fifth chapter is occupied with a discussion of the "Dermis and its Diseases," or the various forms of acute and chronic inflammation of the external meatus, technically known as *otitis*. The ordinary effects of the former affection are resolution, discharge of serum, pus or mucus, or ulceration; of the latter, different secretions, polypoid growths, or caries of the petrous portion of the temporal bone. Each of these topics is treated with great ability, and illustrated by instructive cases. It is well known that the different forms of otitis may extend to the brain, giving rise to softening and suppurations of the cerebral tissues, and deposits of serum, lymph, and pus in the arachnoid sac in the vicinity of the ear.

Such occurrences are most liable to show themselves in chronic otitis in scrofulous subjects, and in persons of a broken-up constitution, laboring under marked disorder of the general health. Hence, such affections require the closest vigilance on the part of the practitioner, and yet it is melancholy to think how much they are generally neglected. When the brain or its membranes are threatened, the proper treatment is, of course, leeching and blistering, with other revulsive remedies, and the usual general antiphlogistic means. No chronic discharge of the ear should ever be disregarded. The idea that the disease upon which it depends will gradually disappear of its own accord, or that the patient, to use a vulgar and unmeaning expression, will gradually "out grow it," can not be too severely reprehended. No conscientious or intelligent surgeon will ever act upon such a principle; he knows it to be wholly false, and that it has been the cause of an immense amount of deafness and also of the loss of many lives.

*Polyps*, or polypoid growths, are not unfrequently met with in the ear, being generally the result of long-continued irritation of the dermoid layer of the meatus, to the surface of which they are usually attached, sometimes by a narrow pedicle, at other times by a broad base. Mr. Toynbee mentions that these bodies occasionally take their origin in chronic inflammation of the mucous membrane of the tympanum, or in obstruction of the Eustachian tube. The latter occurrence can of course happen only when the closure of the tube leads to chronic disease in the meatus or in the tympanic lining or cavity, in both of which latter situations the author has observed the morbid products in question.

Polyps of the ear are always attended with discharge, usually more or less profuse and persistent, and at times, if not generally, excessively offensive. Some degree of deafness is commonly present; the patient often complains of dizziness and confusion in the head, and the suffering is always increased in damp states of the atmosphere. A careful inspection never fails to determine the nature of the affection, even in its earlier stages.

The author divides these growths into three classes: the raspberry cellular polyp, the fibro-gelatinous polyp, and the globular cellular polyp. Of these, the first is the most frequent, and is so called because the numerous round heads of which it consists, and which are attached by small filaments to a central stem, give its surface somewhat the appearance of that of a raspberry. It is very soft, friable, vascular, and of a deep-red color, varying in volume from that of a mustard-seed to that of a body so large as to fill up the whole meatus, beyond which it sometimes projects. A more suitable name for this polyp than the one given it by the author, would be vascular or granular.

The fibro-gelatinous polyp resembles, in its general appearance, the gelatinous polyp of the nose. It may attain the size of the last joint of the thumb, and is generally attached to the meatus by a single narrow root, while its body, which is generally somewhat globular, is often studded with several offsets or younger growths. It is of a white jelly-like hue, and is essentially composed of a cellulo-fibrous structure, interspersed with watery fluid.

The globular cellular polyp was first described by Mr. Toynbee, who considers it as essentially different from the preceding. It is usually of small size, of a deep-red color, softer than the ordinary polyp, globular in shape, and perfectly smooth on the surface. Confined to the inner fourth or sixth of the meatus, to the upper part of which it is commonly attached, it hangs down like a curtain, wholly or partially concealing the tympanic membrane, and occurs chiefly in children or growing persons; its distinguishing characters being founded upon its deep-red appearance, its situation, and the nature of the accompanying discharge, which always contains flocculi of mucus, like small particles of thread.

The treatment of these several classes of tumors varies. For the destruction of the raspberry polyp, our author prefers the use of the lever-ring forceps, an instrument devised by himself; or where this is inapplicable, the use of the potassa cum calce, or Vienna paste. The various astringent remedies he considers as entirely inefficient, both in preventing growth and in diminishing discharge. For the removal of the fibro-gelatinous polyp the best resource is extraction, escharotics exercising no beneficial impression upon its peculiar texture. A small pair of ring forceps is the most suitable instrument for the operation. The treatment of the globular cellular polyp is very simple, the growth being usually readily destroyed by the persevering employment for a week or a fortnight of some astringent lotion, as a solution of acetate of lead, zinc, alum, or tannin; a few drops of the fluid being poured into the meatus several times a day, and a slight discharge, meanwhile, maintained over the mastoid process, with a view to the prevention of congestion in the middle ear.

Mr. Toynbee speaks of osseous and molluscous tumors of the external meatus; their occurrence, however, is extremely rare, and they need not therefore detain us here. Of the molluscous growth of the ear, Mr. Toynbee states that he has several specimens in his museum, and he illustrates his description with some beautiful drawings. The morbid mass sometimes extends to the brain, causing death.

Chapter eighth comprises an admirable account of the structure and functions of the *tympanic membrane*, based essentially upon the author's personal observations, and accompanied by a large number of original drawings, executed in exquisite style. The next chapter treats of the diseases of this membrane, discussing successively its epidermoid, dermoid, and fibrous layers, the consideration of the mucous lining being postponed till the affections of the tympanic cavity come under review. Although replete with the most valuable information, interspersed with instructive cases and splendid wood-cuts, the want of space compels us to forego the pleasure of offering any comments. We cannot, however, avoid giving the subjoined extract in reference to relaxation of the fibrous laminæ of the tympanic membrane, comprising, as it does, a most excellent account of this rare disease, in an exceedingly small compass:—

"The *causes* of this disease are: first, the effects of an ordinary cold, producing hypertrophy of the mucous layer; second, inflammation of the fibrous layers. From either of these causes, the membrana tympani may lose its natural degree of resiliency and become flaccid so as to fall inward, and approach more nearly to the promontory than is natural—a change which ends in great dullness of hearing. This dullness may, however, be temporarily relieved by pressing out the drum to its natural position, either by swallowing with closed nostrils, by attempting a forcible expiration, or by forcibly and rapidly inhaling air through the nose. No sooner, however, is the act of swallowing in the natural way repeated, than the air escapes from the tympanic cavity, the membrana tympani falls inward, and the dullness immediately returns. The *treatment* depends in a measure upon the cause of the affection. If the mucous membrane lining the tympanum be thickened, counter-irritation over the mastoid process should be practiced; if the fibrous laminæ are inflamed, leeches should be applied to the margin of the meatus. Where the fibrous laminæ are weakened, a solution of the nitrate of silver applied to the outer surface of the membrane is frequently of great service. There are cases where the deafness is not in the least degree relieved by forcing air into the tympanic cavity: when this happens, it is most probable that partial anchylosis of the stapes has taken place."

*Perforation* of the tympanic membrane from ulceration and other affections, and the use of an artificial substitute, are the principal topics discussed in chapter tenth. The most common cause of this accident is scarlet fever, but it is also frequently produced by strumous disease; and the invariable result is a diminution of the hearing power, the degree of the impairment depending upon the size of the opening. Such an affection, however induced, is necessarily incurable; but it is consolatory to know that the deafness occasioned by it often admits of great relief, as was shown long ago by Itard and Deleau, and more recently by Todd, Yearsley, and the author of the work before us. The treatment originally employed consisted in the introduction into the bottom of the meatus of soft lint and cotton-wool, gently moistened with water. Deleau had a patient who was greatly benefited by wearing, in this way, the central piece of an onion. The cotton-wool, however, is the article usually preferred, and the improvement from its use is frequently most striking; but, in order to insure its success, the substance should be kept continually moist, otherwise it will cease to become a conductor of sound.

After various researches upon the function of hearing, the results of which were communicated to the Royal Society, and the performance of experiments upon persons deaf from perforation of the tympanic membrane, Mr. Toynbee came to the conclusion that an artificial membrane might be constructed that would answer the purpose of the natural tympanic membrane much better than cotton-wool or linen; and the substance upon which he finally settled is a piece of vulcanized india-rubber, about three-quarters of an inch in diameter, interposed between two very fine plates of silver, not more than three-fourths of a line in diameter, a silver wire of sufficient length being attached to the outer one to admit of the easy introduction and withdrawal of the apparatus. "The artificial membrane is of the greatest benefit in those cases in which there is a well-defined aperture in the natural membrane, or where, that structure being entirely absent, there is simple hypertrophy of the mucous membrane of the tympanum, with or without discharge from its surface." The author adds that he has employed it advantageously in many hundred cases. He very properly cautions against its premature use, or before the complete disappearance of the inflammation causing the perforation. The artificial membrane should be carefully cut to the size and shape of the natural, and should at first be worn only a few hours a day, and that not in contact with the remnants of the tympanic membrane, lest its pressure should serve as a source of irritation. In all cases the apparatus should be removed at night, and if there be any discharge, the parts should be freely syringed morning and evening.

FIG. 1.

In chapter eleventh, the author, after having discussed the anatomy of the *Eustachian tube*, proceeds to speak of its various lesions, in the course of which he describes the proper method of exploring this canal, with the aid of a contrivance of his own, originally pointed out by him in a paper read before the Medico-Chirurgical Society of London as early as 1853; his investigations having been prompted by the fact that he had found the Eustachian catheter, held in such high esteem by Kramer and some other aurists, in great measure, if not wholly, useless as a means of diagnosis. This contrivance he calls the *otoscope*, (*Fig.* 1.) It consists of an elastic tube about eighteen inches long, each end of which is tipped with ivory or ebony. The manner of using the instrument is thus described by the author:—

"One end of it is to be inserted into the ear of the patient, and the other into that of the medical man, who must take care that no portion of the tube touches any neighboring body. When the patient swallows a little saliva, the mouth and nose being closed, if the Eustachian tube be pervious, at the moment that he feels a sensation of fullness in the ear, the surgeon will hear most distinctly a faint crackling sound, produced apparently by a slight movement of the membrana tympani. This crackling sound is that most usually heard;

Fig. 2.

but in some instances, where the mucous membrane of the tympanum is thick, a gentle flapping sound will be detected in its place. If in a case of suspected obstruction of the Eustachian tube, the otoscope fail to reveal any sound during the act of deglutition; if no sound be heard when the patient makes a forcible attempt at expiration with the mouth and nose tightly closed; and if the history of the case, the symptoms and appearances, agree with those already laid down as appertaining to obstruction of the Eustachian tube, I think the surgeon is justified in affirming that the tube is obstructed, and has no need of the use of the Eustachian catheter."

In regard to the Eustachian catheter, at one time so much in vogue, and still employed by certain surgeons, the author thinks that its use is very limited, being restricted chiefly to the examination of the tube, and the inflation of air, and he prefers altogether what he calls the *explorer*, (*Fig.* 3,) a contrivance of his own, consisting of an elastic tube, a foot and a half long, one end of which has a flat mouth-piece of ivory, with a few deep notches in it, while the other is provided with a small steel nozzle, adapted to the further extremity of the catheter, which is not quite as large as an ordinary crow-quill. The catheter having been inserted into

FIG. 3.

the Eustachian tube in the usual way, the surgeon holds it with the left hand, and places one end in his mouth, and the other in the catheter, grasping it also with the left hand. "With his right hand thus at liberty, the surgeon is now to take the *otoscope* and introduce one end of it into the

FIG. 4.

ear of the patient, who may hold it there, the other end being held by the surgeon in his own ear; or the tube may be made so tight as to remain there without being held, leaving the operator's right hand still free." See *Fig.* 4.

"The medical man next proceeds to blow air gently through the explorer, at the same time that he listens through the otoscope to ascertain whether the air enters the ear, and if it does, what is the peculiar sound it produces. When the tympanum is unobstructed by mucus, the air is heard to pass in a stream against the inner surface of the membrana tympani, but when mucus is present, a peculiar gurgling is heard; and if the mucous membrane itself is thickened, a peculiar squeak or bubbling is also perceptible. It is not advisable to blow with force into the ear, but rather to make a few gentle successive puffs, attentively listening during each, to detect the kind of sound that may be heard in the tympanum."

Of the operation of *puncturing* the tympanic membrane, devised by Sir Astley Cooper, Mr. Toynbee expresses, as a general rule, an adverse opinion, declaring that it is seldom required or useful, and that, if not judiciously performed, it is liable to produce the most injurious consequences. It is well known that it signally failed in nearly every instance in the hands of the great English surgeon.

Two long chapters, comprising seventy-two pages, with numerous illustrative cases and drawings, are allotted to the history and treatment of the lesions of the *cavity of the tympanum.* The principal topics discussed under this head, besides the purely anatomical, are the diseases of the mucous membrane, such as the various forms of inflammation, congestion, ulceration, rigidity, the formation of bands of false membrane, anchylosis of the stapes to the fenestra ovalis, and disconnection of the incus and stapes.

The *mastoid cells* next receive their share of attention. Their anatomy is considered first, and then their diseases, the latter being subdivided into those of childhood and those of adult life. Both classes of these affections are liable to eventuate in caries and necrosis of the bone, in lesion of the brain and its membranes, and in paralysis of the portio dura.

Fatal disease of these cells may be induced, first, by purulent infection, arising from the entrance of pus into the circulation through the lateral sinus, and, secondly, by an extension of the morbid action to the cerebellum or its membranes. The former of these occurrences was first noticed by Dr. Abercrombie, in his work on the brain, but it has since been more fully described by Watson, Bruce, Wilde, and Gull, particularly the latter, whose papers in *Guy's Hospital Reports* have thrown much light upon the subject.

Chapter fifteenth treats of the nervous affections of the ear, or of what is usually termed nervous deafness. Under this head are included, first, those affections in which the ear alone is involved; secondly, those in which both the ear and brain are concerned; and, lastly, those which consist in ulceration of the membranous labyrinth, and in caries and necrosis of the petrous portion of the temporal bone.

*Malignant disease* of the ear is uncommon. The author thinks that it usually originates in the mucous membrane lining the cavity of the tympanum, from which it gradually extends outward through the auditory tube to the outer orifice, where it is apt to be mistaken for polypous growths, at the same time that it encroaches upon the whole of the surrounding parts. Assuming at one time the character of fungous hematodes, and at another of encephaloid, it occurs at all periods of life, though more commonly in young than in elderly subjects; the ages in three cases detailed by Mr. Toynbee being, respectively, three, eighteen, and thirty-five. Its progress is generally very rapid, and the issue of course always fatal.

The chapter on the *deaf and dumb* is treated with the author's accustomed ability and good practical sense. "The number of deaf-mute children," says he, "examined, and from whom the facts in this chapter were obtained, amounted to 411. Of these, 313 were congenital cases, and 98 were the effects of different diseases acquired subsequent to birth." This statement affords a better idea than anything else of the immense labor bestowed upon the preparation of this portion of the work. We subjoin the following practical hints relative to the proper mode of examining children supposed to be deaf and dumb, as there are very few practitioners who are familiar with the subject:—

"From the absence of precise experiments from which accurate conclusions could be drawn, great difference of opinion frequently exists, even among medical men, as to whether a child suspected of being deaf and dumb really is so.

"It frequently happens, therefore, that a child is reported not to be deaf, because it always starts or looks up when the door of the room is loudly knocked, or the floor over the room is tapped with considerable force, or the fire-irons in the room are permitted to fall, or the piano is played. A similar opinion is often formed because a child can utter some short syllables, as 'Ma,' 'Pa,' etc., it being argued that no child could learn to utter these sounds unless it had heard them. It is also asserted that a child could not have been born deaf, because the defect was not discovered until it had reached the age of a year and a half or two years.

"In reply to the above arguments in favor of a child's being able to hear, it must be borne in mind that loud sounds are always accompanied by more or less vibration of the walls and floor of the apartment, which can be *felt* by a person whose attention may thus be attracted, although totally deaf. A child may also learn to utter short words by simply imitating the movements of the lips of the parent, or nurse, without the exercise of the sense of hearing. The plan adopted by me to ascertain whether a young child is deaf, consists, in the first place, of allowing it to sit on the knee of the nurse or parent, and be amused by something, and then while its eyes are fixed upon the object, to speak loudly, or shout, taking especial care that the breath does not reach the patient. Again, let the child, its attention distracted as before, be placed with its back toward the surgeon, who should, when near it, clap his hands loudly, ring a large bell, or blow a powerful whistle, always taking care that his own shadow is not seen, and that the child is screened from the movements of the air, while the nurse is warned not to start or suddenly look up; or the surgeon may come into a room, the door of which has been some time open, and where the child is seated with its back toward him surrounded by toys, and perform similar experiments. If the child does not evince any symptom of hearing, by suddenly lifting up its eyes, turning round, or starting, it must be concluded that it is wholly deaf; but if, on the contrary, it looks up each time the surgeon shouts, or turns round quickly the instant the hands are clapped, it is evident that some power of hearing exists, and steps should be taken to ascertain the extent of such power, and how far it may enable the child to be orally taught."

The last chapter in the book is on *ear-trumpets* and their use; and, although very short, abounds in excellent suggestions concerning this important subject.

In bringing our analysis of Mr. Toynbee's labors to a close, we should be doing injustice to our feelings if we were to withhold from him our cordial acknowledgments for the pleasure and profit we have derived from the examination of his excellent treatise. The work, as was stated at the outset of our notice, is a model of its kind, and every page and paragraph of it are worthy of the most thorough study. Considered all in all—as an original work, well written, philosophically elaborated, and happily illustrated with cases and drawings—it is by far the ablest monograph that has ever appeared on the anatomy and diseases of the ear, and one of the most valuable contributions to the art and science of surgery in the nineteenth century.

## Figures and Tables

**Fig. 1. f1:**
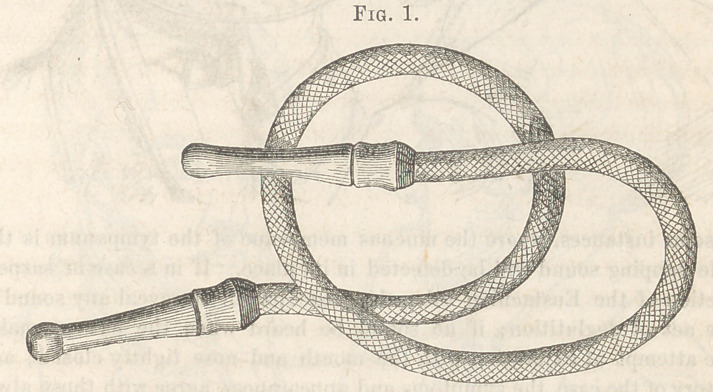


**Fig. 2. f2:**
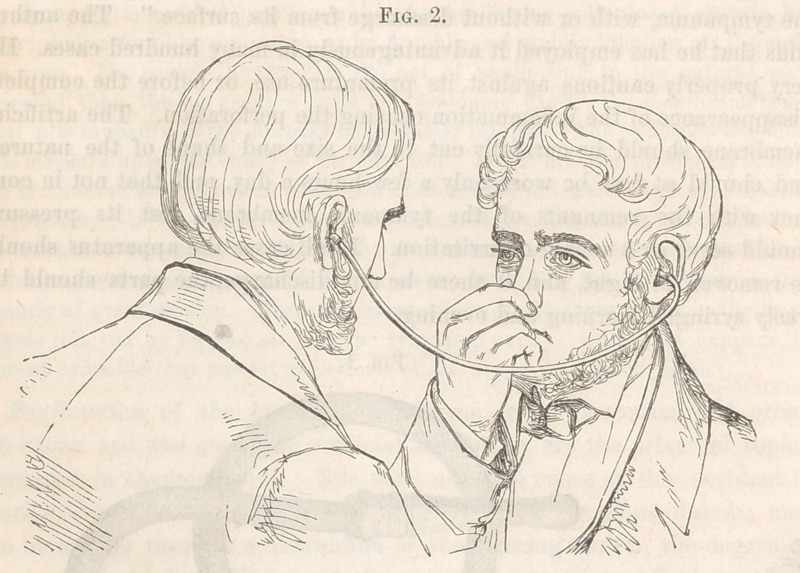


**Fig. 3. f3:**
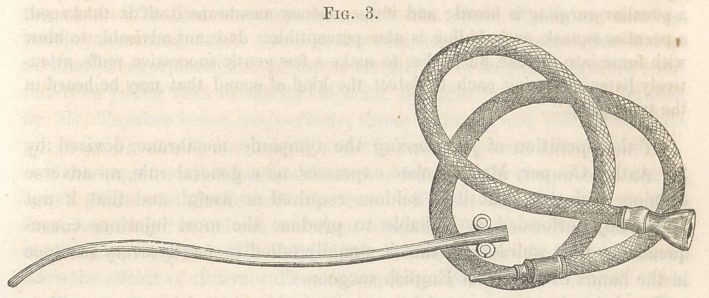


**Fig. 4. f4:**